# The role of NK cells in HIV-1 protection: autologous, allogeneic or both?

**DOI:** 10.1186/s12981-016-0099-6

**Published:** 2016-03-18

**Authors:** Jef Hens, Wim Jennes, Luc Kestens

**Affiliations:** Immunology Unit, Department of Biomedical Sciences, Institute of Tropical Medicine, Nationalestraat 155, 2000 Antwerp, Belgium; Department of Biomedical sciences, University of Antwerp, 2000 Antwerp, Belgium

**Keywords:** HIV-1, Natural killer cells, KIR, HLA, Protection, Allogeneic

## Abstract

Natural killer (NK) cells specialize in killing virally infected- or tumor cells and are part of the innate immune system. The activational state of NK cells is determined by the balance of incoming activating and inhibitory signals mediated by receptor-ligand binding with the target cell. These receptor-ligand bonds mainly consist of the killer immunoglobulin-like receptors (KIR), which are expressed at the cell surface of NK cells, and their ligands: the highly variable human leukocyte antigen -class I molecules (HLA). Absence of an inhibitory receptor-ligand bond lowers the NK cell activation threshold, whereas an activating receptor-ligand bond stimulates the cell, potentially overcoming this threshold and triggering NK cell activation. NK cells influence the course of infection as well as the acquisition of HIV-1. Several lines of evidence relate the activating NK cell receptor KIR3DS1, in the presence or absence of its putative ligand HLA-Bw4, with slower disease progression as well as resistance to HIV-1 infection. Overall, resistance to HIV-1 infection predominantly correlates with activating KIR/HLA profiles, consisting of e.g. activating KIRs, group B haplotypes, or inhibitory KIRs in absence of their ligands. Such a conclusion is less evident for studies of HIV-1 disease progression, with studies reporting beneficial as well as detrimental effects of activating KIR/HLA genotypes. It is likely that KIR/HLA association studies are complicated by the complexity of the KIR and HLA loci and their mutual interactions, as well as by additional factors like route of HIV exposure, immune activation, presence of co-infections, and the effect of anti-HIV-1 antibodies. One newly discovered NK cell activation pathway associated with resistance to HIV-1 infection involves the presence of an iKIR/HLA mismatch between partners. The absence of such an iKIR/HLA bond renders donor-derived allogeneic HIV-1 infected cells vulnerable to NK cell responses during HIV-1 transmission. Therefore, theoretically, HIV-1 would be eliminated before it has the chance to infect the autologous cells in the recipient. While this “alloreactive” NK cell mechanism is especially relevant to HIV transmission in monogamous couples, it would be interesting to investigate how it could influence resistance to HIV in other settings. The objective of this review is to summarize the knowledge about these autologous and alloreactive NK cell responses with regard to HIV-1 outcome.

## Background

HIV-1 is considered to be one of the most widespread viruses, with 37 million people globally living with HIV-1 in 2014 and prime endemic areas situated in South and East Sub-Saharan Africa [1]. Nonetheless, the sexual transmission efficacy of HIV-1 is remarkably lower compared to other viruses (0.01–0.001 %) and is influenced by a variety of viral, immunological, physical and behavioral factors. Especially the innate immune response in the genital mucosa seems to affect the HIV-1 transmission efficacy, as it is capable of inducing a swift antiviral immune response against both free and cell-associated viruses (reviewed in [2]). A successful infection by HIV-1 is mostly established (in 80 % of all HIV-1 infections) by the transmission of a single viral clone, which exposes a weakness of HIV-1 transmission [3]. Therefore, an immune response targeting these clones is more likely to prevent infection compared to other stages in HIV-1 transmission or infection.

Natural killer (NK) cells are part of the innate immune system and are considered to be the main virally infected- and tumor cell killing units of this branch of the immune system. Furthermore NK cells are also present as resident cells in the vaginal, uterine and gut mucosa; forming a rapid first line of defense against incoming pathogens (reviewed in [4]). Accordingly, NK cells are associated with protection against a variety of viral infections including HIV-1. In order to develop a better understanding of the resistance pathways where NK cells may play a significant role, an adequate study population is required. In this respect, HIV-1 exposed seronegatives (ESN) comprise a population with remarkable resistance to HIV-1 transmission, despite being constantly at risk.

NK cells are displayed as promising mediators of HIV-1 protection. Studies examining ESNs or slow progressors linked the beneficial effect with certain key features of NK cell activation, the killer immunoglobulin-like receptor (KIR) on NK cells and its ligand the human leukocyte antigen-class I molecules (HLA) on the target cells. Differences in KIR/HLA associations related to resistance to HIV-1 (HIV-1 resistance) or disease progression accentuate the complexity of interactions with HIV-1 infected target cells [5]. Furthermore, NK cell-mediated HIV-1 resistance was dependent of the HIV-1 donor during sexual transmission, suggesting a role for NK cell responses against “non-self” or “allogeneic” target cells [6].

### Natural killer cells

One of the protagonists of the innate immune response is the natural killer (NK) cell, phenotypically characterized by its expression of CD56 and CD16 on the cell membrane [7]. Based on this expression NK cells can either be labelled “cytotoxic” (CD56^dim^ NK cells), predominantly producing perforin and granzyme B; or “immune-regulatory” (CD56^bright^ NK cells), secreting IFN-γ, TNF-α, IL-10, IL-13 and GM-CSF [8, 9]. This NK cell functionality is coordinated by the balance of incoming activating and inhibitory signals upon encounter with a target cell. During this encounter the signals originate from a variety of receptor/ligand bonds with the target cell [10–17]. NK cells receive inhibitory signals through inhibitory KIRs (iKIRs) (characterized by a long (L) cytoplasmic tail) and CD94-NKG2A. Activating signals are received through activating KIRs (aKIRs) (characterized by a short (S) cytoplasmic tail), as well as natural cytotoxic receptors (NCR) (NKp30,-44,-46), CD94-NKG2C,-E or NKG2D. KIRs can be further subdivided by the number of extracellular domains: 2D or 3D. The inhibitory KIR3DL1 receptors bind human leukocyte antigen (HLA)-A and –B molecules carrying the Bw4 epitope [18]; while KIR2DL1/2/3 receptors bind HLA-C molecules with either a C1 or C2 signature [19]. Despite reasonable homology between activating and inhibitory KIRs, to date, only KIR2DS1 has been shown to have the same ligand as its inhibitory counterpart, although with lower affinity [20]. Recently however, an HIV-1 peptide-dependent functional competent bond was demonstrated between KIR3DS1 and the Bw4 ligand HLA-B*57:01 [21]. With this, a potential vital piece of evidence is delivered for the existence of a KIR3DS1-HLA-Bw4 bond, as was predicted by epidemiological studies but remained presumptive by the lack of proof [22–24]. The necessity for unconventional, virally-derived peptides in the peptide binding groove of KIR3DS1 to mediate a bond suggests a similar pattern for the other aKIR-HLA bonds, clarifying the current lack of proof for these bonds [22].

**Fig. 1:**
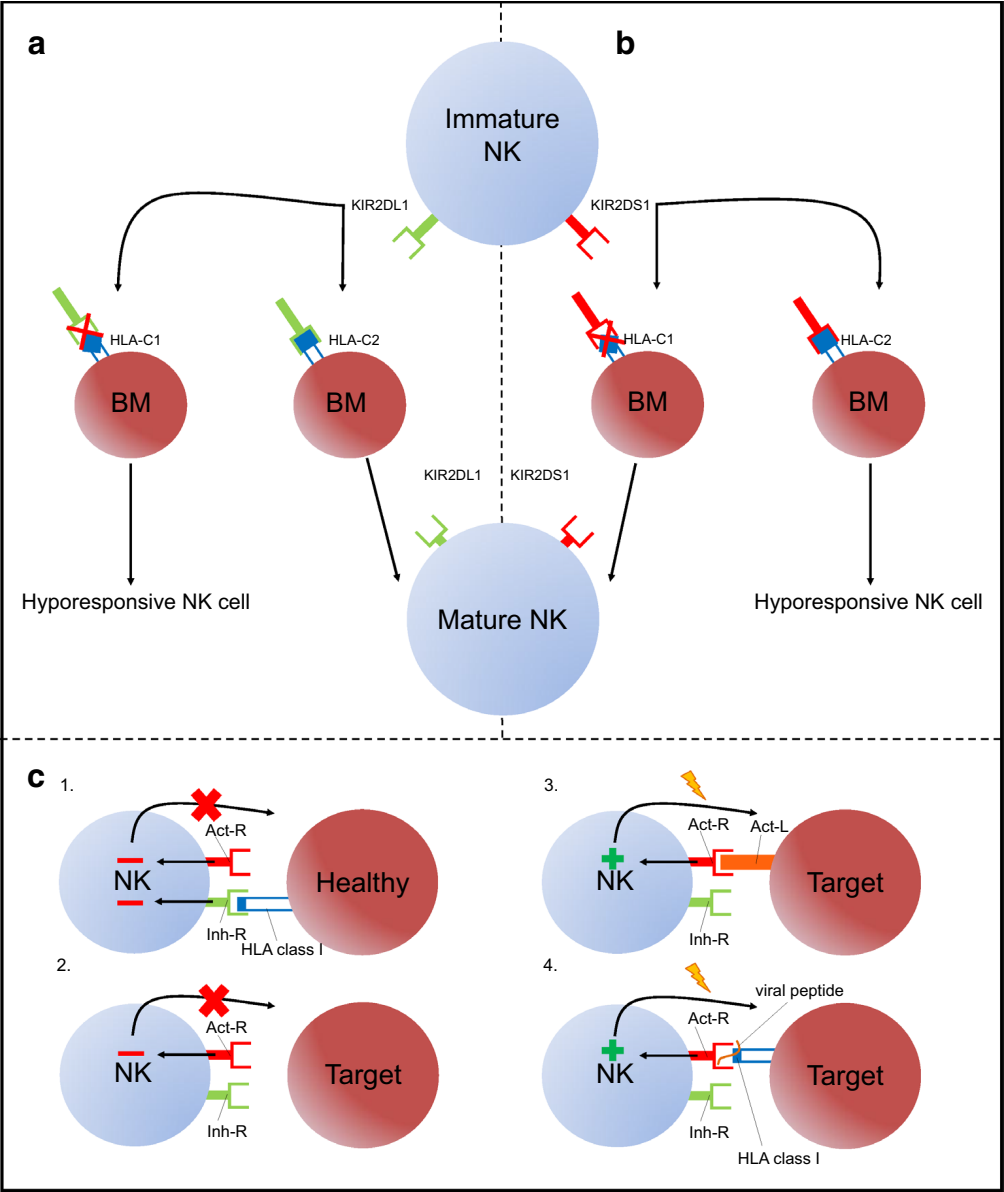
NK cell education and activation pathways. NK cell education can be mediated by iKIRs (KIR2DL1) as well as aKIRs (KIR2DS1) expressed at the immature NK cell membrane. In case of education by iKIR (**a**), a bond between iKIR and self-HLA is necessary to develop fully functional NK cells, whereas its absence abrogates NK cell education resulting in hypo-responsive NK cells. In contrast, in aKIR mediated education (**b**) is the absence of the aKIR-ligand necessary for the licensing of immature NK cells, whereas its presence will generate hypo-responsive NK cells. KIR/HLA interactions also play a pivotal role in the activation of NK cells (**c**). Tolerance (*red minus*) can be mediated by the presence of an inhibitory receptor (Inh-R) -HLA bond and the absence of an activating impulse (*red minus*) (**c**.*1*.) or solely by the absence of an activating signal (**c**.*2*). However in the absence of the Inh-R-ligand bond (“missing self”) an activating NK cell receptor (Act-R) binding non-HLA class I ligands (Act-L) (“induced self”) suffices to induce a cytotoxic NK cell response (*green plus*) (**c**.*3*). NK cell activation is also provoked in the presence of viral/tumor peptides in the HLA class I binding groove of target cells, benefitting the HLA class I-binding affinity of aKIRs (“altered self”) at the expense of iKIRs (“missing self”) (*green plus*) (**c**.*4*)

Activation of NK cells is directed by the balance of incoming inhibitory and activating signals (Fig. 1c). More specifically, mature NK cells in the absence of their self-HLA ligand (missing self) lowers their activating threshold and become sensitive for the presence of activating ligands (induced or altered self) expressed by virally infected or tumor transformed target cells. Alternatively, activation of NK cells can also be achieved when the magnitude of the activating signal(s) overwhelms the dominant inhibitory signal [25]. Further research on the “missing self” model revealed a mechanism called “licensing” or “education”, which takes place during NK cell maturation and profoundly influences the functionality of the matured NK cell. This licensing process allocates functionality towards NK cells capable of creating a bond between iKIR and self-HLA during maturation (Fig. 1a). Absence of such a bond will result in incompetent NK cells also called “hypo-responsive NK cells”. These “rules” will preserve tolerance towards healthy, self-HLA expressing cells, while ensuring NK cell responsivity towards peptide-altered or self-HLA deficient virally-infected or transformed target cells [26–32]. Activating KIRs, by contrast, were not known to play a role in NK cell education, up until Fauriat et al. [33] demonstrated an inverse NK cell education mechanism directed by KIR2DS1 (Fig. 1b). Licensing of KIR2DS1^+^ NK cells was paradoxically only attained in the absence of the corresponding HLA-C2 ligand, whereas presence of the HLA-ligand generated hypo-responsive NK cells. This newly identified education mechanism complements the inhibitory counterpart in preventing autoimmunity and in recognition of altered HLA class I molecules by NK cells [34, 35]. Of note, education of NK cells does not result in lifelong functionality, but will constantly adapt to the present cellular environment, meaning that NK cells who were once hypo-responsive can become functional again and vice versa [36, 37].

## Autologous NK cell activity against HIV-1 infected cells

### Influence of HIV-1 infection on NK cell activity

#### The presence of the CD56neg NK cell subset during HIV-1 infection

In healthy subjects, NK cells are divided into two subsets: CD56^bright^ (~10 % of total NK cell population) and CD56^dim^ (~90 % of total NK cell population) NK cells; though a third subset is present in negligible frequencies: the dysfunctional CD56^neg^ NK cell subset [38]. During acute HIV-1 infection, a pathological NK cell redistribution from CD56^dim^ towards CD56^neg^ NK cells is perceived, accompanied by aberrant NK cell functioning [39]. The decrease in cytotoxic capabilities of CD56^neg^ NK cells is the result of the reduced expression of activating receptors (aKIRs and NCRs), an increase in expression of inhibitory NK cell receptors and reduced secretion of cytokines (IFN-γ, TNF-α and GM-CSF) [40–42]. NK cells are affected by high and chronic viremia, which impairs their antiviral functioning, ultimately contributing to the disease progression [39]. This redistribution is directly associated with viral replication, despite the inability of HIV-1 to infect NK cells. Suppression of viral replication by anti-retroviral therapy is associated with restoration towards the normal NK cell subset distribution, suggesting that the impact of HIV-1 on NK cell distribution could be reversible [43]. In vitro/vivo administered IL-2 has also been shown to contribute to the restoration of NK cell subset distributions [39, 44].

#### HIV-1 impaired dendritic cell functioning limits NK cell cytotoxicity

During acute HIV-1 infection, NK-dendritic cell (DC) crosstalk could be of vital importance for producing an efficient and competent immune response by amplifying the inflammatory potency of the innate and subsequently the adaptive immunity. Nevertheless, HIV-1 has a negative impact on both these cell types. The expanding CD56^neg^ NK cell subset reduces the capacity of NK cells to lyse immature DC (iDC) in order to avoid deficient T cell priming, a process called “DC-editing” [39, 45]. CD56^neg^ NK cells also have a reduced production of IFN-γ and TNF-α, which are necessary for DC maturation [39, 40]. HIV-1 infection directly impacts DC functioning as they produce less IL-12, -15 and -18 during acute HIV-1 infection, leading to less IFN-γ production by NK cells which retrospectively results in poor DC maturation. However, DC cytokine production can be restored in HIV-1 patients after receiving anti-retroviral therapy [39]. Finally, the chronic phase of HIV-1 infection is characterized by an increased secretion of IL-10. IL-10 induces the generation of tolerogenic DCs using direct (by modulating maturation) [46] and indirect (by switching DC editing from iDC to matured DC) [47] mechanisms. In summary, HIV-1 infection impairs NK and DC function and, as a consequence, their crosstalk; leading to feeble, non-specific and aberrant immunity.

#### HIV-1 uses accessory proteins to escape NK cell recognition

HIV-1 impairs NK cell function and escapes recognition by the expression of accessory proteins. These intracellular proteins (in)directly disturb NK cell activity by affecting a wide variety of immunological pathways. The HIV-1 protein “Nef” is able to down-regulate HLA class I molecules on the surface of infected cells [48]. Downregulation of HLA class I molecules may prevent recognition by CD8^+^ T-cells, but might render these cells vulnerable for NK cell mediated lysis by absence of an iKIR-HLA bond. However, Nef evades NK cell activation by selectively sparing KIR ligands HLA-C and HLA-E, while mainly the CD8^+^ T-cell receptor ligands HLA-A and to a lesser extent HLA-B are downregulated [49–51]. In other words, Nef-induced HLA class I downregulation results in the partial evasion of T- and NK cell recognition of HIV-1. The viral proteins Tat and Vpu are also able to downregulate HLA class I molecules, though be it by the blockage of distinct steps in the synthesis of HLA molecules [52, 53]. Furthermore, these viral proteins can also undermine NK cell functioning by denying activation through activating receptors [54–56] and impairing crucial interactions between DC and NK cells [57, 58]. Clearly, the HIV-1 accessory proteins dampen the virally infected phenotype of the host cell and try to mimic the behavior of normal healthy cells in an attempt to avoid recognition by the immune system, including NK cells. In this way HIV-1 manages to fool both the primary and the secondary line of viral defense with only a few proteins.

### NK cell activity during HIV-1 infection

During acute HIV-1 infection, DC-secreted cytokines (IFN-α and IL-15) activate, arm and expand the NK cell population in an attempt to constrain viral replication by NK cell mediated killing [59]. HIV-1 developed multiple evasion strategies (mentioned above) which impair NK cell activation. This feature of HIV-1 indicates that the pressure exerted by NK cells threatens the survival of the virus [60]. This is illustrated by the many associations between slower progression towards the acquired immune-deficiency syndrome (AIDS) in slow HIV-1 progressors and certain characteristics of NK cells. These characteristics are mostly related to improved NK cell recognition, which directly influences NK cell activity. Multiple examples highlighting the pressure and influence that NK cells exert on HIV-1 infection will be listed in the following paragraphs (Table 1).

**Table 1:** KIR and HLA genotypes related to HIV-1 outcome

HIV-1 outcome	Receptor/ligand		Mechanism	Ref
HIV-1 resistance	KIR3DS1/S1	HLA-Bw4	No/less inhibition and increased activation, promoting faster NK cell activation	[85, 86]
	KIR3DS1/x	HLA-Bw4	No/less inhibition and increased activation, promoting faster NK cell activation	[87]
	KIR3DS1/L1		Higher activating ratio, stronger NK cell response	[84]
	KIR3DL1*h	HLA-B*57	“Amplified” education, resulting in stronger NK cell response in absence of ligand	[72]
	KIR3DL1	Bw4	Educated KIR3DL1+ NK cells had increased anti-HIV-1 ADCC mediated cytotoxicity against allogeneic target cells	[76]
	KIR3DL1	HLA-Bw6/Bw6	Absence/lowering of inhibitory threshold, promoting faster NK cell activation	[88]
	KIR2DL2/3	HLA-C2/C2	Absence/lowering of inhibitory threshold	[88]
	KIR2DS4del		Unknown	Unpublished
	KIR2DL1	HLA-C2	Absence of HLA-C2 expression by allogeneic target cells induces cytotoxicity based on KIR/HLA mismatch	[6]
	Haplotype B/x		Multiple aKIRs inducing stronger response	[6, 84–88]
Slower disease progression	KIR3DS1	HLA-Bw4-80I	Slower disease progression; Robust expansion during early infection; Inhibition in vitro viral replication	[59–61]
			Increase of copy numbers is associated with inhibition of viral replication	[62]
			In vitro production of viral inhibiting chemokines (CCL3-5), preventing HIV-1 entry	[123]
	KIR3DS1	HLA-B*57/58	Lower CD38-expression, increased degranulation and IFN-γ production	[64]
	KIR3DS1		Associated with higher CD4+ T-cell counts	[63]
	KIR3DL1/S1	HLA-Bw4	KIR3DL1-dose dependent-licensed NK cells exert cytotoxicity via KIR3DS1-mediated activation	[66]
	KIR3DL1	HLA-Bw4	Increased polyfunctionality by KIR3DL1 licensed NK cells	[65]
			Educated KIR3DL1+ NK cells had increased anti-HIV-1 ADCC mediated activation	[75]
	KIR3DL1*h	HLA-Bw4-80I	Delayed progression to AIDS, increased degranulation, TNF and IFN-γ production	[66, 67]
	KIR3DL1*h	HLA-B*57	“Amplified” education, resulting in strong NK cell response, increased NK cell trifunctionality	[66, 68, 69]
			In vitro production of viral inhibiting chemokines (CCL3-5), preventing HIV-1 entry	[123]
	KIR3DL1*004	HLA-Bw4	Absence/lowering of inhibitory threshold, because of intracellular expression of KIR3DL1	[66]
	KIR2DL3	HLA-C1	Lower viral load and higher CD4 count associated with HIV-1 specific NK cell responses	[73]
	KIR2DL4		CD4+ T-cell preservation, higher copy number resulting in increased IFN-γ production in SIV-infection	[124]
Rapid disease progression	KIR3DS1	HLA-Bw4-80I	Rapid progression, no education of KIR3DS1 expressing NK cells	[80]
	KIR3DS1(/S1)		Rapid progression, robust immune activation accelerating disease progression	[59, 80]
	KIR2DS2/3		Rapid progression, robust immune activation accelerating disease progression	[80]
	KIR2DS4*001		High viral load and accelerated HIV-1 transmission, immune activation accelerating disease progression	[82]
	KIR2DL2/3	HLA-C1	Higher viral load and increased mortality	[83]
	Haplotype B/x		Rapid progression, robust immune activation accelerating disease progression	[80, 81]

Summarization of the KIR, HLA or KIR-HLA haplo-/genotypes associated with HIV-1 resistance and disease progression towards AIDS. HLA class I genotypes associated with altered HIV-1 outcome are not included as its function is not solely relevant for NK cell responses but also for CD8^+^ T cell responses. Additional data in Table 1 obtained from [123] and [124]

#### NK cells influencing HIV-1 disease progression

As NK cell activation is mainly controlled by KIR and HLA molecules, it is plausible that certain KIR and HLA alleles would be associated with a slower disease progression towards AIDS. The following paragraph will discuss the potential of KIR3DS1,-L1,-2DL1-4 receptors and anti-HIV-1 antibodies (Ab) in improving disease outcome.

##### The influence of activating receptors

The intra-individual genetic combination KIR3DS1/HLA-Bw4-80I (HLA-Bw4 antigen with isoleucine at position 80) is well-known for its association with slower disease progression [59]. Evidence of improved in vitro viral suppression and expansion during acute primary HIV-1 infection of KIR3DS1^+^ NK cells in HLA-Bw4-80I bearing individuals supports the beneficial effect of KIR3DS1^+^ NK cells on HIV-1 progression [60, 61]. However, the actual pathways used to activate and license these NK cells are still point of debate. As mentioned previously, the binding of KIR3DS1 with HLA-Bw4 is not fully proven and activation might depend on viral peptides bound by HLA-Bw4-80I [21]. In analogy to the KIR2DS1-licensing pathway (mentioned above) [33], where the presence of KIR2DS1 with corresponding ligand HLA-C2 will not result in licensing, functional-competent KIR3DS1^+^ NK cells would therefore have to be licensed by co-expressing iKIRs [62]. In contrast, the sole carriage of KIR3DS1 was also associated with slower disease progression. In this situation, KIR3DS1^+^ NK cells could be educated to their full competence, in resemblance with the KIR2DS1-licensing pathway [63, 64]. In conclusion, it is clear that KIR3DS1+ NK cells dampen HIV-1 progression, though the activation and licensing mechanisms remain unclear.

##### The influence of inhibitory receptors

The inhibitory counterpart of KIR3DS1, KIR3DL1, is characterized by highly polymorphic alleles which leads to high variability in the expression of KIR3DL1. HIV-1 slow progressors and elite controllers who possess KIR3DL1 alleles resulting in high expression (KIR3DL1*h) and HLA-Bw4-80I had increased NK cell functionality [65–69]. The enhanced NK cell functionality seen in the presence of these KIR3DL1*h alleles can be explained by another feature of licensing supported by the “rheostat model”. The intensity of licensing is dose-dependent, in which an increased amount of iKIR-self-HLA bonds will lead to stronger licensing and result in a stronger reaction in its absence [70, 71]. Additionally, co-expression of an KIR3DL1*h allele with HLA-B*57 not only slowed disease progression, but also induced a protective effect against HIV-1 acquisition [72]. Licensed KIR2DL1-3 NK cells were also associated with slower disease progression as they largely contributed to NK cell functionality and were seen to expand in early HIV-1 infection [73]. Specific HIV-1 peptide NK cell responses were largely mediated by licensed KIR2DL3^+^/C1^+^ patients with the support of a more activating oriented KIR phenotype (B haplotype) [74]. Similar results were previously described in Hepatitis C virus (HCV) infection [75]. The HIV-1/HCV protective effect can be explained by the low binding affinity of KIR2DL3 for HLA-C1, compared to KIR2DL2, resulting in a lower threshold for activating ligands to overrule inhibition subsequently leading to NK cell activation. Lastly, anti-HIV-1 Ab are able to guide NK cells towards target cells by binding to the Fc-receptor CD16 on NK cells. This bond also provides an additional activating signal, which contributes to overcome the inhibitory threshold [76]. Anti-HIV-1 Ab improve the HIV-1 defensive capacities of NK cells which was found to result in a lower viral set point and a slower disease progression [77, 78]. The actions of anti-HIV Ab in aiding NK cell cytotoxicity can be grouped and named as antibody-dependent cell cytotoxicity (ADCC) [79–82].

##### Accelerated disease progression

In contrast to the aforementioned beneficial genotypes, certain KIR and/or HLA genotypes were also seen to accelerate disease progression. KIR3DS1, both with [83] and without [59] HLA-Bw4-80I has been associated with accelerated disease progression. Likewise, other aKIRs [83–85] were also related to a detrimental HIV-1 outcome. Similarly, Ebola infected patients possessing the aKIR genes KIR2DS1 and -3 were associated with fatal outcome [86]. It is possible that NK cells with an activating KIR profile would preferably generate a pro-inflammatory environment, compared to more inhibitory “tolerable” NK cells. We suggest that this pro-inflammatory environment will contribute to chronic immune activation and attraction of target cells, accelerating disease progression. Recent findings also associated co-carriage of KIR2DL3 and HLA-C1 with higher viral load and increased mortality rates [87]. Although this was previously seen to be favorable in HIV-1 and HCV infection, it is in line with the previous findings as activation defined NK cells are more plausible to accelerate disease progression [74, 75].

#### NK cell functioning in HIV-1 resistance

In the search for HIV-1 resistance mechanisms, ESN are often investigated as population of interest. ESNs are exposed to HIV-1 but remain seronegative, suggesting that they are resistant to HIV-1 infection. Functional studies of a Vietnamese ESN cohort first demonstrated an increase in activated NK cells [88]. Genetic epidemiological studies of African ESN cohorts later found activation oriented KIR/HLA genotypes, consisting of KIR B haplotype and iKIRs in the absence of their ligands (KIR2DL2/3-HLA-C2 and KIR3DL1-HLA-Bw6) to be associated with resistance to HIV-1 ([89] and later confirmed in ref [6]). The authors hypothesized that the absence of the inhibitory iKIR/HLA signal would lower the NK cell activation threshold which would result in a faster NK cell activation upon encounter with activating ligands on infected target cells. Similarly, the presence of KIR2DL3 in combination with HLA-C1, resulting in a lower activation threshold and accelerated activation eventually; was associated with slower disease progression [74] as well as HIV-1 resistance [90]. The hypothesis of an accelerated NK cell activation in ESNs was strengthened by epidemiological data on KIR3DS1 (haplotype B) in ESNs. Multiple studies ascribed the protective effect in ESNs to the increased or decreased prevalence of KIR3DS1 and KIR3DL1 respectively [90–93]. The elevated KIR3DS1/L1 ratio seen in these ESN cohorts, however, was not always in association with HLA-Bw4 [90, 91]. These results might rather highlight the beneficial effect of an activating KIR haplotype in general, instead of the specific KIR3DS1-HLA-Bw4 bond. Exceptionally, in an ESN cohort consisting of hemophilia A patients, no KIR or HLA allele was correlated with HIV-1 protection. However, absence of this association could be ascribed to the influence of the far more infectious parental transmission route, potentially overruling the effect of KIR and HLA on transmission which is seen to be correlated with HIV-1 resistance in sexual transmission [94]. In summary, HIV-1 resistance can be related to more “reactive” NK cells, be it by the increase of aKIRs or the lowering of the activation threshold.

#### Interpretation of NK cells influencing HIV-1 resistance and disease progression

In an attempt to order and structure all the information regarding the influence of NK cells on HIV-1 infection and transmission, we will discuss the findings in the previous paragraphs. In multiple in vivo and in vitro studies, the effect of KIR3DS1^+^ NK cells in combination with HLA-Bw4 on HIV-1 infection was seen to result in slower disease progression. These data suggest that these KIR3DS1^+^ NK cells become activated during acute HIV-1 infection, potentially by the presentation of HIV-1 peptides on the HLA-Bw4 molecules. In contrast, KIR3DS1 is also associated with an accelerated disease progression. In addition, data correlating other aKIRs and a lower activation threshold with a detrimental outcome, suggests that the activation oriented KIR profile is responsible for the accelerated rather than a slower disease progression. Though this data is less convincing than the evidence concerning KIR3DS1^+^ NK cells being related to slower disease progression, it is supported by similar results in Ebola infection and should be taken into account when describing NK cell responses in HIV-1 infection. Genetic data of ESNs suggest a beneficial effect of KIR3DS1^+^ NK cell on the induction of HIV-1 resistance. In general, HIV-1 resistance has been associated with NK cells who are prone to activation, by an increased prevalence of aKIR or by the lowering of the activation threshold.

In conclusion, we propose an alternative hypothesis, irrespective of the well-known beneficial KIR3DS1^+^ NK cell effect on disease progression. If the early pro-inflammatory NK cell burst does not suffice to eliminate HIV-1, NK cells in possession of a more activation-oriented KIR B haplotype will paradoxically accelerate HIV-1 spread and disease progression by attracting target cells and contributing to chronic immune activation. On the other hand, NK cell responses in individuals with a more inhibition-oriented KIR A haplotype will be less inflammatory and will be able to dampen HIV-1 spread and disease progression once HIV-1 mediated HLA class I down-modulation tends to take place.

## The benefit of NK cells in haplo-hematopoietic stem cell transplantation

Although debate is ongoing, HIV transmission mediated by cell-associated virus is believed to be more efficient than transmission mediated by free virus. In vitro and in vivo analysis of SIV-transmission efficacy [95–97] confirmed the competence and successfulness of cell-mediated HIV-1 transmission (reviewed in [98]). Together with the presence of mucosal NK cells, the inter-individual HLA class I variability and the multiple HIV-1 resistance associations might suggest a direct NK cell response against incoming allogeneic HIV-infected cells. The effectiveness of these “alloreactive” NK cells is already verified in immunotherapy applied in acute myeloid leukemia and acute lymphoid leukemia: haploidentical-hematopoietic stem cell transplantation (haplo-HSCT).

### The clinical relevance of alloreactive NK cells in haplo-HSCT

A haplo-identical donor and its recipient share one identical HLA-haplotype and differ at the HLA class I and II locus of the other haplotype. In HLA-identical HSCT, T-cells mediate the graft-versus-leukemia (GVL) effect, enhance engraftment and reconstitute immunity. But T-cells in an allogeneic environment become reactive and first need to be extensively depleted from the haplo-HSCT graft to avoid graft-versus-host disease (GVHD). Remarkably, the GVL effect in T-cell depleted haplo-HSCT was handed over to alloreactive NK cells driven by a KIR/HLA mismatch [99, 100]. Also “megadoses” of CD34^+^ stem cells are used for transplantation to promote donor-derived immune recovery and to help overcome graft rejection induced by recipient’s anti-donor cytotoxic T-cells (reviewed in [101]). CD34^+^ stem cell megadoses also create a donor-like derived environment in the recipient, resulting in donor HLA-based NK cell education [27, 102]. To date, NK cells are capable of remaining alloreactive for more than 5 years after transplantation [103, 104].

### Favorable KIR/HLA interactions in haplo-HSCT

NK cell alloreactivity in haplo-HSCT is only guaranteed for licensed ‘missing self’ KIR/HLA combinations specifically consisting of donor inhibitory KIR that recognize HLA allotypes present in the donor but which are lacking in the recipient [102]. In addition, absence of the inhibitory CD94/NKG2A was also necessary as its ligand, HLA-E, is expressed on all HLA class-I expressing cells [99, 103]. The presence of the activating B haplotype in an acute myeloid leukemia cohort was also found to be related to a low risk of leukemic relapse and prolonged survival [105]. Moreover, different aKIRs have proven their value in haplo-HSCT, as KIR3DS1 and KIR2DS2 were associated with mild acute GVHD and decreased leukemic relapse, respectively [106, 107]. KIR2DS1^+^ NK cells have shown to lyse C2^+^ leukemia cells, capable of overriding a threshold composed by different inhibitory signals, decreasing GVHD and redirecting the NK cells towards the lymphe nodes [103, 108–112]. Given these results, KIR2DS1 co-expression on donor-derived NK cells is a major contributor to the alloreactivity of NK cells and is already seen as one of the more important parameters in the calculation of the alloreactive NK cell subset size. Besides killing recipient DCs, alloreactive NK cells also kill residual recipient T-cells, preventing host versus graft (HvG) responses, which results in adequate engraftment [112].

The discovery of alloreactive NK cells as the major allo-effectors in haplo-HSCT is one of the largest breakthroughs in the recent history of treating leukemia. Alloreactive NK cells have the potential to further improve leukemia therapy with less invasive and burdensome techniques. In relation to HIV-1 infection, certain parallels can already be drawn between HIV-1 resistant and haplo-HSCT NK cell characteristics, such as the beneficial effect of an activating B haplotype and the presence of aKIRs. The haplo-HSCT field precedes the HIV-1 field in terms of knowledge about alloreactive NK cell responses. Therefore, it is useful to take these results in regard when investigating alloreactive NK cells and its role in HIV-1 resistance.

## Alloreactive NK cells in HIV-1 infection

A reasonable amount of HIV-1 research is focused on the innate immune system, and more specifically on the relationship of KIR and HLA variability with HIV-1 resistance or disease progression. In most cases only the recipient’s characteristics are taken into account, whereas NK cells are also able to mediate allogeneic responses, as is seen in haplo-HSCT (explained in “The benefit of NK cells in haplo-hematopoietic stem cell transplantation” section). In the following paragraphs, existing evidence of alloreactive NK cells playing a role in resistance to HIV-1 is summarized.

### Alloreactive NK cells in sexual HIV-1 protection

Jennes et al. recently investigated a cohort of HIV-1 discordant couples (dC) from Dakar, Senegal, consisting of one HIV-1^−^ and one HIV-1^+^ partner [6]. The study first of all confirmed known associations of activating KIR genotypes and inhibitory KIRs in absence of their HLA ligands with HIV-1 resistance [89]. In addition, a specific mismatched allogeneic KIR/HLA combination, consisting of recipient KIR2DL1/HLA-C2 with donor HLA-C1/C1, was found increased in the HIV-1 dC group compared to the concordant (i.e. when both partners were HIV-1+) group. Conversely, a specific matched allogeneic combination, consisting of recipient homozygous KIR2DL3 with donor HLA-C1/C2, was found increased in the HIV-1 concordant group compared to the dC group. In theory, the allogeneic KIR/HLA mismatch generates a missing-self context, whereby the expression of ligands for activating NK cell receptors on HIV-1 infected donor cells would induce “alloreactive” responses by recipient NK cells. In contrast, a matched allogeneic KIR/HLA combination contributes to the inhibition of alloreactive NK cell responses. In vitro co-cultures of healthy donor NK cells with CD4^+^ T-cells derived from HIV-1 patients were performed to investigate the magnitude of the allogeneic NK cell responses. A clear increase in CD4^+^ T-cell death was seen in the presence of an allogeneic KIR/HLA mismatch [6]. These results support the hypothesis of the presence of HIV-1 protective alloreactive NK cell responses. Similar in vitro studies with an increased sample size are now being carried out by our research group in an attempt to confirm and further unravel the mechanisms behind these responses.

Alongside KIR and HLA, NK cell mediated ADCC is regarded as an additional mechanism capable of lysing HIV-1 infected CD4 + T-cells [80, 82]. Although HIV-1 ESNs do not possess anti-HIV-1 IgG antibodies, it has been shown that IgG transmission from HIV-1 infected mothers to their children during breastfeeding rendered these children HIV-1 resistant [113]. Recently, anti-HIV-1 antibodies capable of triggering ADCC were detected in the seminal plasma and vaginal fluid of HIV-1 patients [114, 115]. ADCC was preferably generated by educated NK cells expressing KIR2DL1 and HLA-C2 [82], similar to the results of Jennes et al. [6] where the combination of a KIR2DL1/HLA-C2 recipient with an HLA-C1 donor was elevated in the ESN cohort. Therefore we suggest that in the presence of an allogeneic KIR/HLA mismatch (seen in [6]), resulting in a lower activation threshold, anti-HIV-1 Ab can further activate NK cells by binding its activating NK cell Fc-receptor CD16 (seen in [82]). Anti-HIV-1 Ab might preferentially induce NK cell alloreactivity as it is not affected by the HIV-1 impaired expression of activating ligands, guaranteeing a competent signal. Furthermore, these anti-HIV-1 Ab are capable of binding HIV-1 infected cells as well as cell-free virions, providing a complete screening for HIV-1 prior to exposure during transmission.

### Alloreactive NK cells during vertical HIV-1 transmission

HIV-1 infected pregnant women are capable of transmitting the virus to their child during gestation, delivery, or breast-feeding. Mother–child interactions can be seen as allogeneic interactions as the child has only one identical haplotype, haplo-identical, with the mother. In analogy with multiple HIV-1 studies investigating ESNs [6, 89–92], the frequency of activating KIR profiles was significantly increased in non-transmitting mothers compared to transmitting mothers [116]. Conversely, an elevated frequency of allogeneic KIR/HLA matches (infantKIR2DL2/2DL3- motherHLA-C1/C1) was observed among HIV-transmitting mother/infant pairs [117], confirming the increased frequency of the matched allogeneic KIR/HLA combination among Senegalese HIV-transmitting concordant couples [6]. In multiple studies, vertical as well as sexual HIV-1 transmission was found associated with sharing of HLA class I alleles between mother and child or between partners [118–121]. In other words, transmission is most likely to occur when educated NK cells are silenced by a KIR/HLA match, resulting in a more HIV-1 receptive environment.

### Future perspectives

Mucosal NK cells are present in the female reproductive tract, from the uterus to the vagina, as well as the gastro-intestinal tract, and express an immature phenotype (reviewed in [4]). Studying the local maturational, migratory and cytotoxic capabilities of mucosal NK cells during HIV-1 transmission will help to calculate the contribution of mucosal NK cells in HIV-1 resistant immune responses. Macaques are an ideal model for these experiments and were already essential for revealing different in vivo migration routes and immune responses during early HIV-1 infection (reviewed in [122]). In addition, the use of HIV-1 dC couples is very suitable to study the impact of the alloreactive mechanism on HIV-1 acquisition. A decent number of studies on HIV-1 dC cohorts did not investigate NK cell alloreactivity. Data from these studies could possibly be reanalyzed with a renewed focus on allogeneic KIR/HLA relations between both partners. In this way, new data can be generated to add to the small amount of existing information on the role of NK cell alloreactivity in HIV-1 resistance. Also the contribution of anti-HIV-1 ADCC towards NK cell activation is an interesting element that needs to be further explored, specifically in the allogeneic context. Nonetheless, studies focusing on NK cell function in the context of HIV-1 often investigate populations that have a high burden of viral co-infections. Since infections, such as Cytomegalovirus, are known to influence NK cell function, future studies should take this possible limitation into account.

## Conclusion

The HIV-1 epidemic has grown out to be one of the most widespread infectious diseases in the world. However, some individuals seem to resist infection with HIV-1, despite repeated exposure to the virus. Throughout the years, different cohorts have been assembled, consisting of such HIV-1 exposed uninfected individuals. These so-called ESNs constitute an interesting population for the study of HIV-1 protective mechanisms in an attempt to develop HIV-1 preventive or curative therapies. Importantly, HIV appears to be extremely vulnerable to the host immune responses during a brief time frame immediately after sexual exposure to HIV-1. This finding resulted in a rediscovered interest for the innate immunity, with NK cells as potential HIV-1 restrictive effector cells. NK cells have not only been linked to HIV-1 resistance but are also found to influence disease progression, although some results indicate that different and even opposing mechanisms might be involved. Examining the protective effects in HIV-1 dC couples revealed KIR/HLA mismatches between both partners, suggesting an HIV-1 protective mechanism orchestrated by alloreactive NK cells, whose usefulness has already been proven in the treatment of leukemia.

In summary, an increasing amount of evidence seems to suggest an important role for alloreactive NK cell responses in resistance to HIV-1 infection. These responses likely take place during the transmission timeframe where HIV-1 is most vulnerable, before the immunological influence of established HIV-1 infection can take effect. This mechanism likely involves an allogeneic KIR/HLA mismatch which triggers the missing self-mechanism. Still, further research is needed to explore the full potential of NK cells as protective mediators during sexual HIV-1 transmission. Retrospective reanalysis of existing HIV-1 dC cohort data in particular could provide valuable insight into these protective mechanisms. Even so, current findings seem promising and could perhaps 1 day lead to a preventive immunotherapy based on the alloreactive NK cell principle.

## Abbreviations

Ab: antibodies
ADCC: antibody-dependent cell cytotoxicity
AIDS: acquired immune-deficiency syndrome
aKIR: activating Killer immunoglobulin-like receptors
CCR: C-C motif receptor
CD: cluster of differentiation
dC: discordant couples
DC: dendritic cell
ESN: exposed seronegatives
Fc: fragment crystallizable
GM-CSF: granulocyte macrophage-colony stimulating factor
GVHD: graft-versus-host disease
GVL: graft-versus-leukemia
haplo-HSCT: haploidentical hematopoietic stem cell transplantation
HCV: hepatitis C virus
HIV-1: human immunodeficiency virus type 1
HLA: human leukocyte antigen
HvG: host versus graft
iDC: immature dendritic cell
IFN-γ: interferon gamma
iKIR: inhibitory killer immunoglobulin-like receptors
IL: interleukine
KIR: killer immunoglobulin-like receptors
NCR: natural cytotoxic receptors
NK: natural killer cell
TNF: tumor necrosis factor
TRAIL: TNF-related apoptosis-inducing ligand
